# Identification of upper thermal thresholds during development in the endangered Nechako white sturgeon with management implications for a regulated river

**DOI:** 10.1093/conphys/coad032

**Published:** 2023-05-23

**Authors:** Madison L Earhart, Tessa S Blanchard, Phillip R Morrison, Nicholas Strowbridge, Rachael J Penman, Colin J Brauner, Patricia M Schulte, Daniel W Baker

**Affiliations:** Department of Zoology, University of British Columbia, 6270 University Blvd. Vancouver, BC V6T 1Z4, Canada; Department of Zoology, University of British Columbia, 6270 University Blvd. Vancouver, BC V6T 1Z4, Canada; Department of Zoology, University of British Columbia, 6270 University Blvd. Vancouver, BC V6T 1Z4, Canada; Department of Resource Management and Protection, and Biology Department, Vancouver Island University, 900 Fifth Street Nanaimo, BC V9R 5S5, Canada; Department of Zoology, University of British Columbia, 6270 University Blvd. Vancouver, BC V6T 1Z4, Canada; School of Biodiversity, One Health, & Veterinary Medicine, College of Medical, Veterinary & Life Sciences, University of Glasgow, 464 Bearsden Rd, Bearsden, Glasgow G61 1QH, UK; Department of Zoology, University of British Columbia, 6270 University Blvd. Vancouver, BC V6T 1Z4, Canada; Instreams fisheries research, 2323 Boundary Rd Unit 115, Vancouver, BC V5M 4V8, Canada; Department of Zoology, University of British Columbia, 6270 University Blvd. Vancouver, BC V6T 1Z4, Canada; Department of Zoology, University of British Columbia, 6270 University Blvd. Vancouver, BC V6T 1Z4, Canada; Department of Fisheries and Aquaculture, Vancouver Island University, 900 Fifth Street, Nanaimo, BC V9R 5S5, Canada

**Keywords:** Conservation, development, sturgeon

## Abstract

Climate change-induced warming effects are already evident in river ecosystems, and projected increases in temperature will continue to amplify stress on fish communities. In addition, many rivers globally are impacted by dams, which have many negative effects on fishes by altering flow, blocking fish passage, and changing sediment composition. However, in some systems, dams present an opportunity to manage river temperature through regulated releases of cooler water. For example, there is a government mandate for Kenney dam operators in the Nechako river, British Columbia, Canada, to maintain river temperature <20°C in July and August to protect migrating sockeye salmon (*Oncorhynchus nerka*). However, there is another endangered fish species inhabiting the same river, Nechako white sturgeon (*Acipenser transmontanus*), and it is unclear if these current temperature regulations, or timing of the regulations, are suitable for spawning and developing sturgeon. In this study, we aimed to identify upper thermal thresholds in white sturgeon embryos and larvae to investigate if exposure to current river temperatures are playing a role in recruitment failure. We incubated embryos and yolk-sac larvae in three environmentally relevant temperatures (14, 18 and 21°C) throughout development to identify thermal thresholds across different levels of biological organization. Our results demonstrate upper thermal thresholds at 21°C across physiological measurements in embryo and yolk-sac larvae white sturgeon. Before hatch, both embryo survival and metabolic rate were reduced at 21°C. After hatch, sublethal consequences continued at 21°C because larval sturgeon had decreased thermal plasticity and a dampened transcriptional response during development. In recent years, the Nechako river has reached 21°C by the end of June, and at this temperature, a decrease in sturgeon performance is evident in most of the traits measured. As such, the thermal thresholds identified here suggest current temperature regulations may not be suitable for developing white sturgeon and future recruitment.

## Teaser

Nechako white sturgeon have had no recruitment over the last 60 years due to many environmental stressors. We investigated physiological effects of summer river temperatures in white sturgeon embryos and larvae. We identified upper thermal thresholds for multiple traits, suggesting current temperature regulations may not be suitable for white sturgeon.

## Introduction

River ecosystems are extremely sensitive to increases in temperature and other climatic variables associated with climate change (Palmer *et al*., 2008, 2009; Watts *et al*., 2015; Pletterbauer *et al*., 2018). Indeed, the effects of warming are already detectable in fish communities in rivers (Daufresne and Boët, 2007), and projected future increases in air temperature (IPCC, 2021) will only increase the stress on these freshwater ecosystems (Albert *et al*., 2021). However, around the world, many rivers are affected by dams (Grill *et al*., 2019), and although these dams have well-documented negative effects by altering flow regimes, changing sediment composition, and blocking fish passage, they also present an opportunity to help regulate river water temperature through managed release of cool water from reservoirs (Olden and Naiman, 2010; Rheinheimer *et al*., 2015; Chen and Olden, 2017).

In western North America, thermal regulation of rivers through dam releases is often focused on mitigating harm to salmonids (Macdonald *et al*., 2012; Munsch *et al*., 2020; Anderson *et al*., 2022; FitzGerald and Martin, 2022). However, this approach neglects other native, and often imperiled, fish species (Zarri *et al*., 2019). For example, in the Nechako river in British Columbia, Canada, a government mandate requires operators of the Kenney Dam to maintain river water temperatures <20°C in July and August to protect the sockeye salmon (*Oncorhynchus nerka*) during their spawning migration (Levy and Nicklin, 2018; Nechako River, 2022; Summer Temperature Monitoring Program, 2022). This is a critical temperature for the sockeye, and without this protection, high pre-spawn mortality occurs (Levy and Nicklin, 2018). However, there is another critically endangered species inhabiting this river: Nechako white sturgeon (*Acipenser transmontanus*) (Nechako River, 2022). Unfortunately, relatively little is known about the thermal limits of white sturgeon, although what data exist suggest that 20°C may be above optimal temperatures for spawning and early development (Wang *et al*., 1985; Webb *et al*., 1999; Hildebrand *et al*., 2016).

In particular, sturgeon early-life stages during the first 2 months of development are extremely sensitive to external stressors like flow, sediment and temperature (Wang *et al*., 1985; McAdam *et al*., 2005; Baker *et al*., 2014; Boucher *et al*., 2014; Bugg *et al*., 2020; Brandt *et al*., 2022; Yoon *et al*., 2022), and these cumulative environmental changes may contribute to the observed long-term recruitment failures for white sturgeon in the Nechako river. Specifically in this river system, increase in fine sediment like sand, increases in temperature and changes in flow affecting spawning grounds are hypothesized to be the driving factors of failed recruitment. The recruitment failure in the Nechako river has been observed since the 1960s, and as such without supplementation intervention, the population is headed toward extinction (Nechako White Sturgeon Recovery Initiative, 2021). However, river temperature regulation on the Nechako is only required during the July and August migration and spawning season for sockeye salmon, and sturgeon spawn earlier in the summer (May and June in the Nechako River; Hildebrand *et al*., 2016), and thus the timing of current regulations is not likely to adequately protect vulnerable sturgeon embryos and larvae. As such, in this study, our objective was to define suitable temperatures across multiple levels of biological organization in Nechako white sturgeon at two of the most vulnerable and understudied developmental stages: embryos and yolk-sac larvae.

We aimed to identify upper thermal thresholds, or tipping points, above the thermal optimum, at which performance begins to decrease as temperature increases. At these sublethal thermal thresholds, a variety of changes occur across biological levels of organization (Schulte, 2014; Jeffries *et al*., 2018). However, different processes—whether at the level of transcriptional, cellular or the whole organism—may have distinct thermal thresholds influencing their function. Determining the sublethal temperature thresholds for species of conservation concern is critical for developing useful management regimes because performance is impacted long before lethal thermal thresholds are reached, and reduced performance can have consequences for fitness (Schulte, 2014). Thus, distinguishing the lethal and sublethal temperatures for developing white sturgeon survival and physiological performance is critical to informing regulatory practices controlling river temperatures in the Nechako river. This is of particular importance for the more vulnerable life stages in the Nechako river, like the spawning adults, embryos, and larvae because of the failed recruitment observed in this system.

To aid in identifying these thermal thresholds for Nechako white sturgeon embryos and larvae, we measured transcriptional and whole-animal physiological responses after temperature acclimation and acute thermal stress across early development. We incubated developing Nechako river white sturgeon to three different, ecologically relevant, temperatures throughout embryogenesis and yolk-sac absorption: 14, 18 and 21°C. These temperatures were chosen based on previously documented river temperatures: for example, in 2021, Nechako river temperatures were at 14°C in June, during the critical embryogenesis stage and reached 18°C by mid-June during the yolk-sac absorption stage. We additionally chose 21°C to test the efficacy of the summer temperature monitoring program for the white sturgeon because the Nechako river reached 21°C by the end of June in both 2021 and 2022 (Environment Canada, 2022). The monitoring program for sockeye salmon focuses on avoiding temperatures >20°C during July and August (Levy and Nicklin, 2018) and white sturgeon embryos and larvae are experiencing temperatures higher than this before these months. Thus, warm water exposures in this system could be playing a role in the failed recruitment for white sturgeon.

Our objective in this study was to define sublethal and lethal thermal thresholds in white sturgeon embryos and yolk-sac larvae to aid in recommendations for temperature regulation through dam management that may help with conserving the Nechako white sturgeon. We measured whole-animal physiological performance through embryo metabolism, yolk-sac larvae morphometrics, and larvae maximum thermal tolerance (CTmax). To test for differences in cellular performance between developmental temperatures, we measured the transcript abundance of 13 genes in the yolk-sac larvae at rest and after an acute thermal stressor. Genes were selected for their roles in thermal stress, hypoxia and blood oxygenation, energy allocation, and growth, to provide insight into multiple physiological processes that could be affected by exposure to warmer temperatures.

## Methods

### White sturgeon husbandry

On May 25, 2021, gametes were collected from wild-caught male and female white sturgeon at the Nechako White Sturgeon Recovery Initiative (NWSRI) in Vanderhoof, British Columbia, Canada (54.0140° N, 124.0130° W). This population is critically endangered, and as such has limited spawning adult individuals (<100 spawning fish). Due to this endangered status, we collected eggs from two females and milt from three males to create a total of six families (3 half-sib families from each female). Eggs were fertilized at 13°C, within 30 min of collection, and were then immediately spread across six petri dishes (~200 embryos per dish). Embryos from each family were evenly distributed across the petri dishes. Embryos adhered to the petri dishes within 3 min (Earhart *et al*., 2020), and the embryos were then transported back to the Initiative for the Study of the Environment and its Aquatic Systems (INSEAS) facility at the University of British Columbia, fully immersed in coolers supplied with air stones and HOBO temperature loggers (Onset Computer Corporation, Bourne, MA, USA). To maintain temperatures <14°C, cool, dechlorinated water from the NWSRI was added to the coolers when necessary throughout the 10-h transit.

On arrival, embryos in the petri dishes were transferred to an incubator (model: MIR-154, PHCBI), held at 14°C with a 12:12 light cycle. At 1 day post-fertilization (dpf) four of the six petri dishes were moved to a separate incubator held at 18°C, and the next day, at 2 dpf, two of those petri dishes were moved to a third incubator held at 21°C. As such, by 2 dpf, the embryos were in three different temperature treatment groups—14, 18 and 21°C (2 petri dishes per temperature)—and they were held at these temperatures for the remainder of the experiment. This schedule of increasing embryo incubation temperatures over 2 days was chosen to avoid complete mortality from thermal shock because fertilization was conducted at 13°C (Wang *et al*., 1985). Note that this design allowed for equal representation of all families across all developmental temperatures. To allow for appropriate comparisons of developmental rates between treatments, accumulated thermal units (ATU) were calculated (dpf × T°C) throughout the experiment (Rombough, 1985; Jay *et al*., 2020).

All petri dishes were held inside 9-l containers filled with water bubbled with air from an air stone and circulated with a pump to maintain dissolved oxygen levels. After hatch, we added bio-balls to the tanks for substrate, and all larvae were kept in the 9-l containers described earlier. Water quality measurements and ~30% water exchanges were completed twice daily to ensure ammonia did not rise >0.5 ppm throughout the experiment. Temperatures were checked multiple times daily, with temperature in each incubator monitored and recorded via HOBO temperature loggers. Mortalities were recorded daily in each treatment throughout the experiment. All embryos and larvae in this study were reared and sampled under guidelines established by the Canadian Council for Animal Care and approved by the Animal Care Committee at the University of British Columbia under Protocol A19–0284.

### Embryo metabolic rate

**Figure 1:**
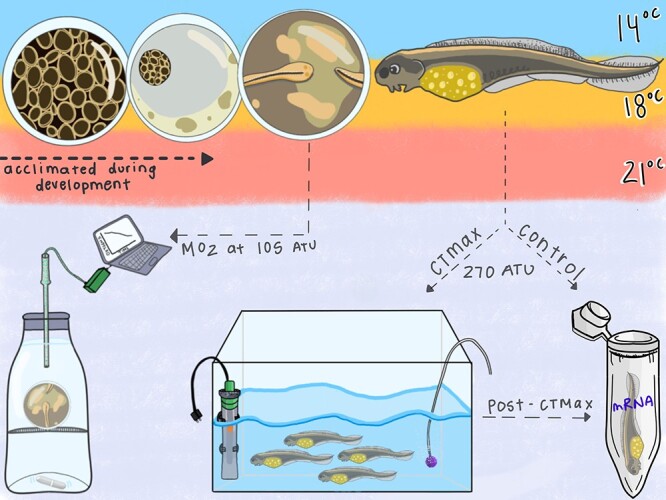
Experimental design for this study. Embryos and larvae were acclimated (in duplicate) to one of three temperatures: 14, 18 or 21°C for the duration of the experiment. Embryo metabolic rate was measured at 105 ATU. Larval critical thermal tolerance (CTmax) was measured at 270 ATU before exogenous feeding. Samples for mRNA measurement were taken from control fish and fish after CTmax for quantification of mRNA gene abundance. Illustration by Madison Earhart.

Measurements of whole-embryo metabolic rate were conducted the day before hatch (105 ATU) for each group (Fig. 1). We chose to use embryo metabolic rate as an indicator of thermal tolerance rather than critical thermal maximum (CTmax) because sturgeon embryos are not completely transparent, making the identification of CTmax end points challenging. Metabolic rate may be a particularly sensitive indicator of thermal challenges in embryos because of their high metabolic demand during development (Dahlke *et al*., 2020). Individual embryos were randomly selected from both petri dish replicates and placed into 1.8-ml custom glass micro-respirometry chambers with magnetic stir bars below a false-bottom mesh to ensure mixing during the trial. All metabolic rate measurements were completed at the embryos’ respective incubation temperature, 14, 18 or 21°C, by placing chambers into an incubator. Three trials were run per day and were conducted between 8 am and 4 pm. After being placed in the metabolic chamber, embryos were allowed to recover for 30 min, to account for handling stress, before the beginning of the metabolic rate measurements. The oxygen probe was placed into the chamber, and then the chamber was sealed with many layers of parafilm to secure the oxygen probe. We assessed metabolic rate through measuring the reduction in oxygen levels using NeoFox fibre optic probes (FOXY system, Ocean Optics, Dunedin, FL, USA). Oxygen levels were recorded every 10 s until air saturation reached 70% inside the chambers (~1.5–2.5 h). After each trial, fish were weighed using a microbalance (Mettler Toledo XPR2). Fibre optic probes were calibrated twice weekly using 100% air-saturated water by vigorously bubbling air into the water and 0% air-saturated water created by the addition of sodium sulfite (1 g/100 ml). Chambers were rinsed with 70% ethanol between trials to kill bacteria, and background oxygen consumption was measured after each trial and found to be negligible (<1% MO_2_). We measured the change in oxygen concentration over time, which was corrected for both the volume of the respirometer and embryo wet mass to give the oxygen consumption rate (μmolO_2_·g^−1^·h^−1^). We chose a coefficient of determination (*R*^2^ value) of 0.9 as our threshold for MO_2_ data (Chabot *et al.*, 2021). A total of nine individual embryos were measured from each acclimation temperature (4–5 individuals from each petri dish replicate).

### Larval morphometric measurements

Length, wet mass, and yolk-sac volume were measured at 113, 162, 213 and 271 ATU after they were euthanized with an overdose of buffered MS-222 (0.5 g·l^−1^). Total length was measured by analyzing photographs, taken through a dissecting microscope at 5× magnification, in ImageJ using the “draw line” tool. All images included a reference ruler (millimetres), and all measurements were taken from the top of the head to the tip of the tail. Wet mass was measured to the nearest 0.000001 g (Mettler Toledo XPR2) after thoroughly drying each individual fish with a Kimwipe. Yolk-sac volume was measured by analysing the dissecting microscope photos on ImageJ by taking measurements of the diameter (YsD) and the length of the yolk-sac (YsL). Yolk-sac volume was then calculated by this formula (Blaxter and Hempel, 1963):

Yolksac volume (YsV) = (π/6)YsL × YsD^2^.

### Critical thermal maximum trials

To assess the maximum thermal tolerance of the yolk-sac larvae, we performed CTmax trials at 270 ATUs for each temperature treatment (Fig. 1). This coincided with the time of yolk-sac absorption and the start of yolk-plug ejection before exogenous feeding. For each trial, 25 individuals were randomly selected from the incubators and placed into a mesh fish breeder net (Hagen, Montreal, Quebec, CA) that was held inside a 20-l experimental bucket containing aerated, circulating, dechlorinated water set to the acclimation temperature of the treatment being tested (14, 18 or 21°C). Sturgeon larvae were held for an hour before the start of the trial to allow time to recover from handling stress (Bugg *et al*., 2020). Trials were run by increasing temperature 0.3°C·min^−1^ with immersed titanium heater sticks (finnex TITANIUM 300+) until the larvae lost equilibrium (LOE) and were unable to right themselves after a disturbance (*i.e.* touching their caudal tail with a blunted probe; Bard and Kieffer, 2019; Yoon *et al*., 2019; Bugg *et al*., 2020; Penman *et al*., 2023). Air saturation was maintained >95% throughout the trial through bubbling air through air stones. Immediately after LOE, the sturgeon was removed from the breeder net and euthanized with an overdose of buffered MS-222 (0.5 g·l^−1^), and whole-body samples were snap-frozen and stored at −80°C until further analysis. At this time, we also sampled control fish (fish not exposed to an acute stressor) from each of the incubation tanks in the same way for mRNA analysis.

### RNA extraction, cDNA synthesis and qPCR

Whole-body total RNA was extracted using an RNeasy kit (Qiagen, Hilden, Germany). For each sample, total RNA concentration and purity were determined using a Nanodrop 2000c (Thermo Scientific). Extracted RNA samples were then stored at −80°C until future use. After extraction, RNA was treated with DNAse to remove any DNA in the sample. Synthesis of cDNA was performed using a qscript synthesis kit (Quantabio, Beverly, MA, USA) with 1 μg of DNAse-treated RNA in a thermocycler (Bio-Rad, Hercules, CA, USA) as follows: 1 cycle of 22°C for 5 min, 1 cycle of 42°C for 30 min, 1 cycle of 85°C for 5 min, followed by a hold at 4°C. After cDNA synthesis, samples were diluted 10-fold with nuclease-free water for subsequent qPCR analysis. cDNA was then stored at −30°C until further use.

Real-time quantitative polymerase chain reactions (RT-qPCRs) for all genes were completed in a total volume of 10 μl per well with Bio-Rad Sso Advanced Universal SYBR-green supermix (5 μl per sample), nuclease-free water (3 μl per sample), primers (100 μM 0.05 μl in 0.95 μl nuclease-free water), and 1 μl cDNA. We measured the mRNA abundance of 13 target genes and two reference genes (Table 1) in whole-body samples, and abundance was normalized to the expression of these two stable reference genes. Reference genes did not differ across different temperature treatments, ATUs or between CTmax and control samples (Chapman and Waldenström, 2015). Primers were designed using a white sturgeon transcriptome (Doering *et al*., 2016) and were assessed for secondary structures and non-target binding. Primer efficiency was determined by generating a 1:10 standard curve for each gene using pooled cDNA. Both no template and no reverse transcriptase controls were run on every qPCR plate, and no contamination was detected. All reactions were completed using an RT-qPCR machine (Bio-Rad CFX96) in a 96-well plate under the following SYBR-recommended cycling conditions: 2 min at 95°C, 40 cycles of 15 s at 95°C and 30 s at 58°C, and melt curves were produced by denaturation for 15 s at 95°C, a decrease for 10 s down to 60°C, and then a gradual increase of 0.5°C·s^−1^ to 95°C. Amplification data were analysed using standard curves created for each gene, and the data for genes of interest were normalized to the geometric mean of expression of the two reference genes (Vandesompele *et al*., 2002; Earhart *et al.*, 2022b).

**Table 1:** Primer sequences for white sturgeon (*A. transmontanus*), *hsp70, hsp90a, hsp90b, hsp47, hif1a, hb-a, hb-b, hxk, pepck-c, pepck-m, g6p, igf2, rps5, rps8.* Target genes were chosen based on their roles in the response to temperature stress, hypoxia, energy allocation and growth. *rps5* and *rps8* were used as reference genes because they showed stable expression across all treatments. Efficiencies are listed as a percentage (%)

Gene name	Gene category	Forward primer	Reverse primer	Efficiency (%)
*hsp70*	Temperature	CCATGAACCCCAGCAACACT	TGCACAACAGAGTCGTCGTA	100.8
*hsp90a*	Temperature	CCTTGATTGCCTCCTCTGTT	GACTCATTCCAACCGCATCTA	103.4
*hsp90b*	Temperature	ACTTGGTCCTTGCTCTCACC	GCGATACCACAGCTCTCAGT	104.9
*hsp47*	Temperature	ACCTGTAATAAGTCCGCATCTC	GTGTACGAACCACCCAAGAA	96.5
*hif1a*	Hypoxia	GCATCTGAGGATAGTGGTAAAG	CTGTTGGCAGTAGGAGAATG	99.6
*hb-a*	Blood oxygenation	CGAATTGTCACCAGGTTCTAT	GTGAGAGGCATCTTTGTAGTT	105.4
*hb-b*	Blood oxygenation	GCTTGCACCAGGGATTT	ACTAAACACCAGCCATCTTAC	104.7
*hxk*	Energy allocation	GAAGCCGCCAGAACAATAA	GTACACCAAGACCCACTTTAG	100.2
*pepck-c*	Energy allocation	CGTATGCCCTGACCTTAATC	CGACAATAACTTGAGACACAATC	100.5
*pepck-m*	Energy allocation	CAGTCAGCGAGTTCGTTTC	CTTTCCAGTGTTCCCAGTATC	97.5
*g6p*	Energy allocation	CGCTCTGCTTCTCCAATAG	CCCTAACAACCTCACACTAAA	98.9
*igf2*	Growth	GGAGAATTACACCAGCAAGAA	CACAGAAAGGACGCCAATAA	95.1
*gh1*	Growth	CGACCGAGTGTTTGAGAAA	AGTGAGCTTCAGCAAAGTAG	104.9
*rps5*	Reference	ACTCGACCCGAATTGGACG	GTTGACTCTACGCAGGGGG	99.9
*rps8*	Reference	GGGCGACCCAATTCATACTT	CCAGGGACAACTGGCATAAA	102.7

### Statistical analysis

Mortality differences between treatments were analysed via Cox proportional hazards model in R v4.1.2 (R core team, 2013) using the “survival” and “survminer” packages (Therneau, 2015; Kassambara *et al*., 2019) to assess the effect of temperature on mortality rate across ATU. To assess the specific differences between temperatures, the “pairwise_survdiff” function from the “survminer” package was applied with a Bonferroni correction.

For all analysis of variance (ANOVA), residuals were analysed with both Shapiro-Wilks and Levene tests to assess data normality and homogeneity of variance. Normality was also assessed by visually inspecting Q-Q plots. If assumptions of either test were violated, data were transformed with a log transformation before the ANOVA; this was only the case for yolk-sac volume data. All ANOVAs were completed in GraphPad Prism 9 with a significance level of 0.05.

Morphometrics (mass, length and yolk-sac volume) were analysed with a two-way ANOVA with acclimation temperature, ATU, and their interaction included in the model as fixed effects. After the ANOVA, multiple comparison tests were performed and corrected with a Tukey honest significant difference (HSD) test.

Embryo metabolic rate data and larval CTmax data were analysed with a one-way ANOVA followed by a multiple-comparison test and corrected with a Tukey HSD test. Acclimation response ratios were also calculated for CTmax data across acclimation temperatures by subtracting the average CTmax of the 14°C acclimation treatment from 18°C (or the CTmax of 18°C from 21°C) and dividing that by the change in acclimation temperature between treatments:

Acclimation response ratio (ARR) = (CTmax18°C − CTmax14°C) / Δ°C

Two different statistical analyses for mRNA abundance were completed. First, a two-way ANOVA was completed for each gene, with acclimation temperature, CTmax and their interaction as the fixed effects. We additionally performed a principal component analysis (PCA) using the “factomineR” (Lê *et al*., 2008) and “factoextra” (Kassambara and Mundt, 2021) packages in R. Data were subset into two groups: (1) mRNA levels at control and (2) mRNA levels following CTmax to analyze how yolk-sac larvae transcriptional responses differed following only acclimation and after an acute thermal stressor.

## Results

### Mortality

**Figure 2:**
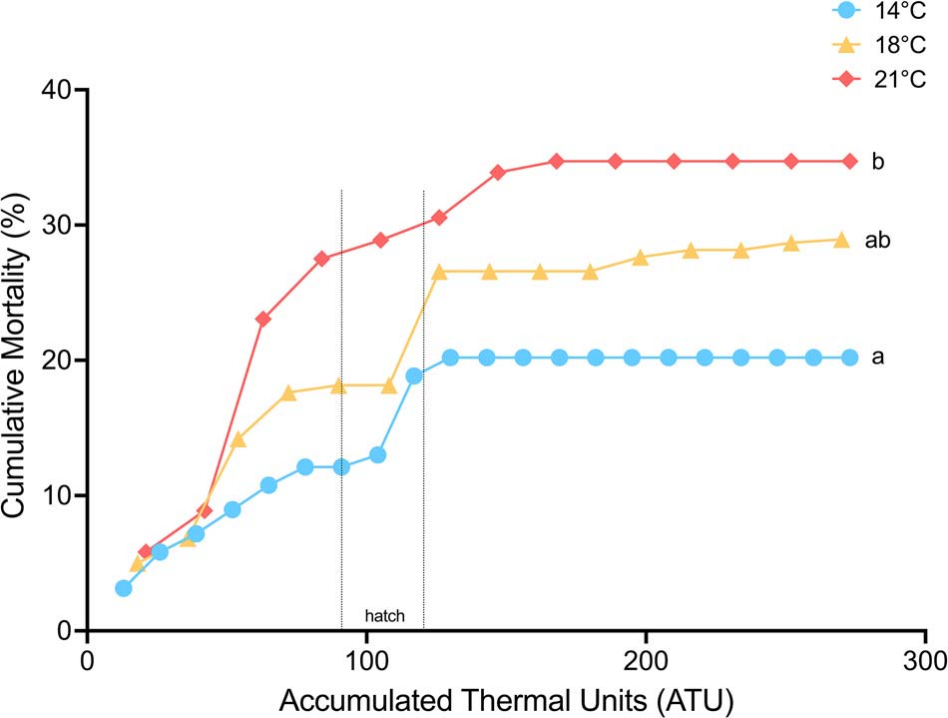
Cumulative mortality (%) of white sturgeon (*Acipenser transmontanus*) embryos and yolk-sac larvae throughout early development. Acclimation temperatures are represented by different colors, 14°C in blue, 18°C in yellow and 21°C in pink. Time of hatch is indicated by the dashed vertical lines on the figure. Letters represent significant differences between acclimation temperatures (*P* < 0.05, Cox proportional hazards model). Data are expressed as percentage cumulative mortality from time of fertilization to yolk-plug ejection (0–280 ATU; *n* = 400–450; 2 petri dishes per temperature each containing all families).

Mortality was significantly impacted by temperature as determined by a Cox proportional hazards model (Fig. 2). Almost all mortality, regardless of acclimation temperature, was observed before hatch around the time of neurulation. However, at 21°C, there was significantly more mortality when compared with 14°C (15.9 ± 3.48% vs 28.2 ± 3.8%; *P* < 0.0001), and 18°C exhibited an intermediate level of mortality (21.9 ± 0.04%), which was not significantly different from either 14 or 21°C.

### Embryo metabolic rate

**Figure 3:**
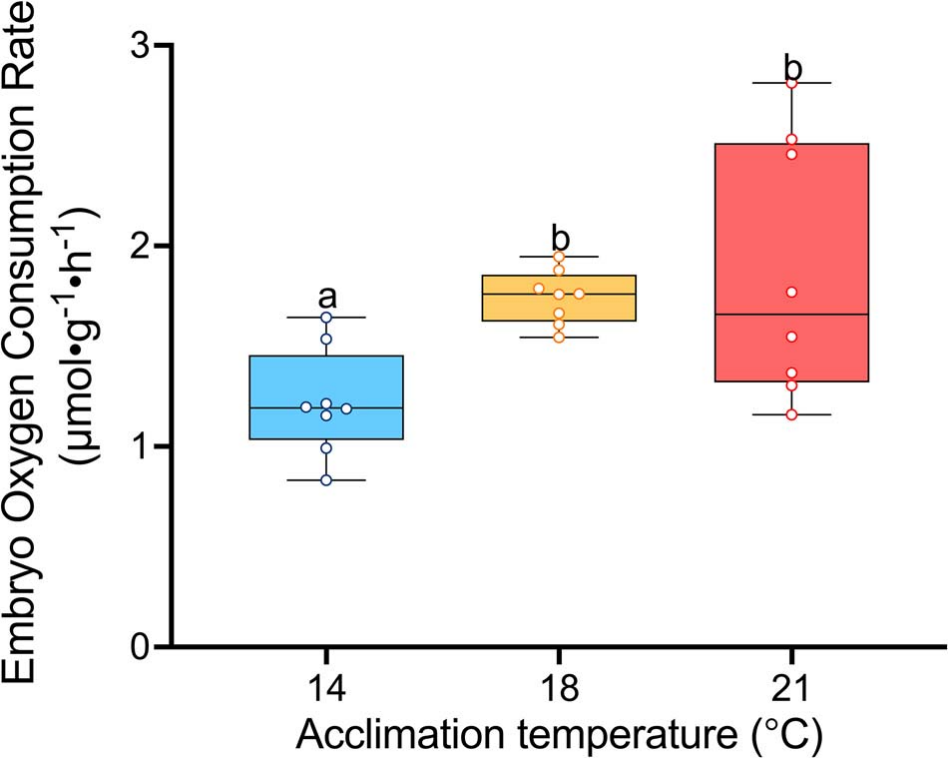
White sturgeon (*A. transmontanus*) embryo oxygen consumption rate*,* acclimated to three different temperatures (14°C – blue, 18°C – yellow and 21°C—pink). Measurements were conducted the day before hatch in each treatment (105 ATU). Letters that differ represent significant differences between acclimation temperatures. Data are expressed as median with quartiles and individual data points are shown (*n* = 8).

One-way ANOVA revealed a significant effect of acclimation temperature on embryonic metabolic rate (Fig. 3; *P* = 0.01). Both the 18- and 21°C-acclimated embryos had a higher metabolic rate than the 14°C-acclimated group, but there was no difference between the 18 and 21°C groups, suggesting a thermal limitation of embryo metabolic rate.

### Larval morphometrics

**Figure 4:**
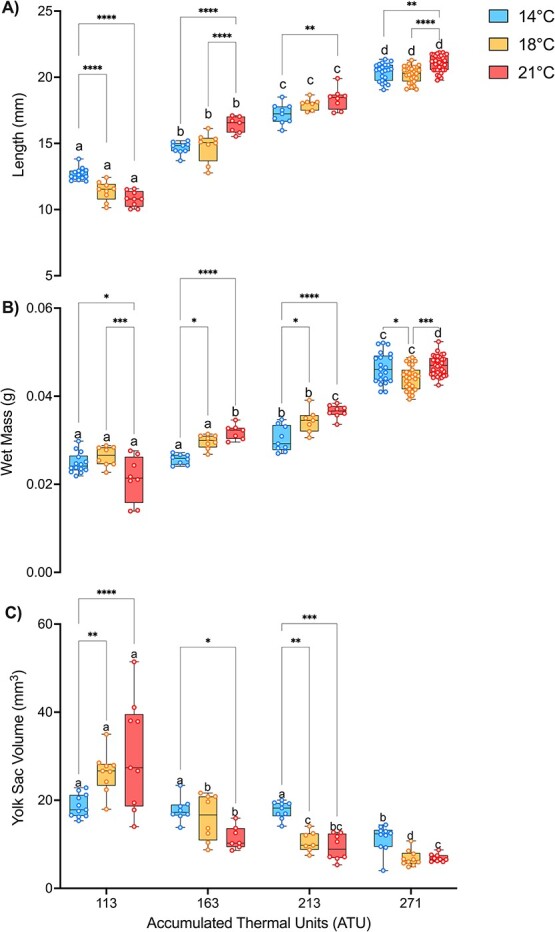
Morphometrics of larval white sturgeon (*A. transmontanus*) acclimated to three different temperatures (14°C – blue, 18°C – yellow and 21°C—pink) across ATUs. Panel A is length, panel B is wet mass and panel C is yolk-sac volume. Asterisks represent differences between temperatures within ATUs. Letters that differ represent significant differences across time within an acclimation temperature. Data are expressed as a median with quartiles and individual data points are shown (*n* = 7–33).

All morphometric data were analysed via two-way ANOVA. White sturgeon larval length was significantly affected by acclimation temperature (Fig. 4a; *P* = 0.0006), ATU (*P* < 0.0001) and their interaction (*P* < 0.0001). Similarly to length, two-way ANOVA revealed a significant effect of acclimation temperature (*P* = 0.0003), ATU (*P* < 0.0001) and their interaction (*P* < 0.0001) on wet mass of larval sturgeon Fig. 4b), and yolk-sac volume (Fig. 4c; *P* < 0.0001) was affected by the same factors. Sturgeon sampled from each temperature grew over time; however, the 21°C larvae were larger than both 14 and 18°C groups at each ATU sampling time with the exception of 113 ATU. In addition, yolk-sac volume was reduced in the 21°C larvae, suggesting that it was more quickly depleted compared with the 18 and 14°C larvae. This suggests that the 14 and 18°C acclimation groups used less energy from the yolk because they maintained more of their yolk-sac size across ATUs.

### Larval CTmax

**Figure 5:**
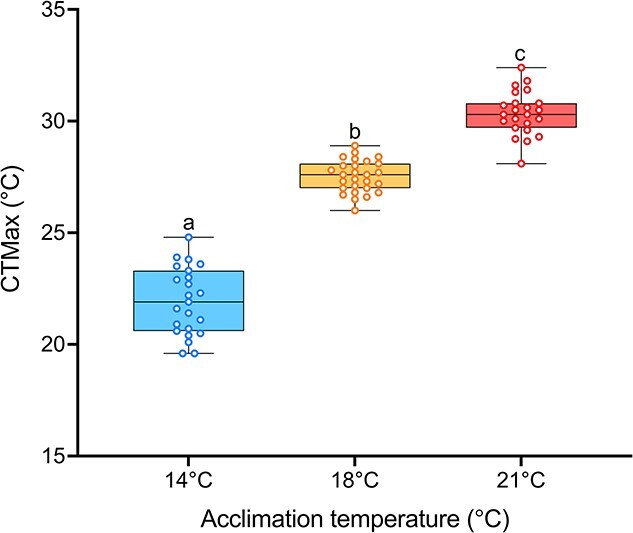
White sturgeon (*A. transmontanus*) larval CTmax at three different acclimation temperatures (14°C – blue, 18°C – yellow and 21°C—pink). Measurements were conducted at the start of yolk-plug ejection in each treatment (270–273 ATU). Letters that differ represent significant differences between acclimation temperatures. Data are expressed as median with quartiles and individual data points are shown (*n* = 23–27).

Maximum thermal tolerance was significantly affected by temperature (Fig. 5; *P* < 0.0001) as detected by one-way ANOVA. The CTmax temperatures were 21.9, 27.5 and 30.4°C for the 14, 18 and 21°C acclimation groups, respectively. The acclimation response ratio for the yolk-sac larvae was quite impressive, with an ARR of 1.4 between the 14 and 18°C acclimation groups and an ARR of 0.9 between the 18 and 21°C group. These results indicate that for each degree change in acclimation temperature, there was about a degree (°C) increase in CTmax.

### mRNA transcript abundance

**Figure 6:**
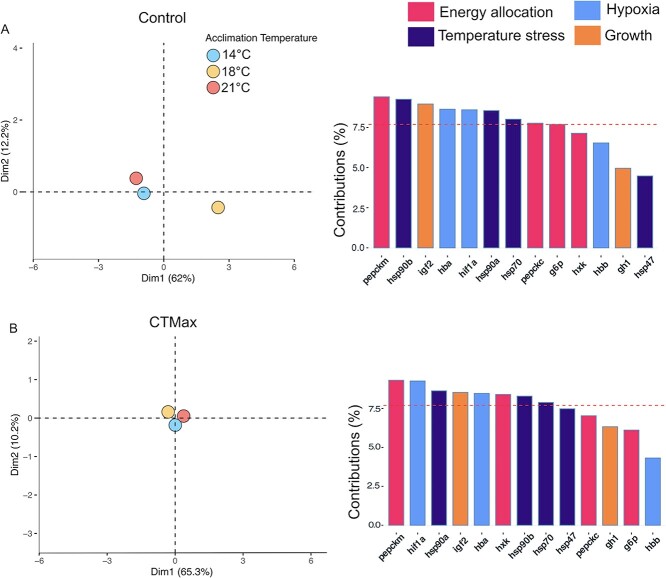
PCA of mRNA abundance of larval white sturgeon (*A. transmontanus*) acclimated to three different temperatures (14°C – blue, 18°C – yellow and 21°C—pink). Panel A is a PCA of mRNA levels and the genes that contribute to the PCs in acclimated and control fish. Panel B is a PCA of mRNA levels and genes that contribute to the PCs in acclimated fish after CTmax trials. Gene contribution figures are colored by different gene function: energy allocation (pink), temperature stress (dark purple), hypoxia and blood oxygenation (blue) and growth (orange). The red dashed line on both gene contribution figures indicates the default average contribution expected for each gene to the overall observed variation.

In the PCA for control larvae mRNA data, principal component 1 explained 62% of the observed variation in mRNA abundance, whereas principal component 2 explained 12% of the variation. The main gene contributions for PC1 and PC2 (principal component; Fig. 6a; S2) were *pepck-m, hsp90b, ifg2, hba, hif1a, hsp90a* and *hsp70.* The PCA for the control mRNA data highlighted separation between the 14 and 18°C acclimation group, with the 18°C fish shifting to the right on principal component 1 (Fig. 6a). However, at 21°C, the fish were more similar to the 14°C fish, suggesting a thermal threshold at 18°C, with cellular performance beginning to decrease at 21°C. In the PCA for CTmax larvae, mRNA data principal component 1 explained 65% of the variance and principal component 2 explained 10%. The genes contributing the most to PC1 and PC2 were *pepck-m, hif1a, hsp90a, igf2, hba, hxk, hsp90b* and *hsp70* (Fig. 6b; S2)*.* Examination of the CTmax principal component analysis revealed a different pattern (Fig. 6b), with all three temperature treatments inducing similar responses to acute thermal stress. These data suggest there were more differences in the mRNA transcriptional response between fish at rest from exposure to warm temperatures and that these fish respond similarly when faced with thermal stress. In both groups, *pepck-m* is contributing the most differences identified between acclimation groups. However, the rest of the genes contributing to observed variation differ between groups at rest and after CTmax. For example, *hsp90b* contributes more at the control point, whereas *hif1a* drove more of the variation in response to CTmax.

**Figure 7:**
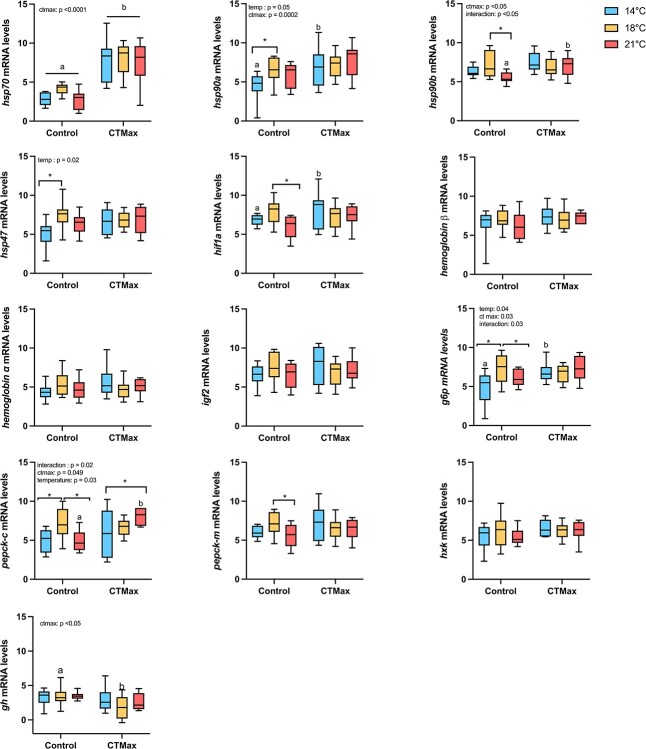
White sturgeon (*A. transmontanus*) larval mRNA levels for all genes measured after acclimation to three different temperatures (14°C – blue, 18°C – yellow and 21°C—pink) at control and after CTmax. Significant differences between acclimation temperatures at control or after CTmax are denoted by an asterisk. Significant differences between control and CTmax measurements within an acclimation temperature are denoted by letters. Significant two-way ANOVA effects are listed on the top of each individual gene panel.

mRNA abundance of each gene was also compared between treatments (two-way ANOVA), and those data are reported in the supplemental material (Fig. 7). Briefly, in the control sturgeon, most genes showed an inverted U-pattern of mRNA abundance levels, where transcription increased from 14 to 18°C but decreases at 21°C. This pattern suggests a thermal threshold in mRNA transcription and cellular performance during development across the biological processes we measured. There were a few genes that were affected by CTmax (Fig. 7) as determined by ANOVA, including *hsp70, hsp90b, g6p* and *pepck-*c (*P*-values are reported in S1). Interestingly, the only gene that increased expression in all temperature groups after CTmax was *hsp70*; in older white sturgeon, we typically observe an increase in additional *hsps* after acute thermal stress (Earhart *et al*., Unpublished data; Penman *et al*., 2023).

## Discussion

In this study, we highlight multiple thermal thresholds across different levels of biological organization for developing Nechako white sturgeon. Typically, non-linear response patterns to increasing temperature represent sublethal thermal thresholds and show an inverted U-shape trajectory (Jeffries *et al*., 2018), and this is demonstrated here across multiple phenotypes as temperatures surpassed 18°C. Development in 21°C had sublethal and lethal effects on both white sturgeon embryos and yolk-sac larvae. After only 2 days at 21°C, embryo metabolic rate was suppressed, demonstrating an inability of white sturgeon to maintain metabolic rate at high temperatures during embryogenesis. There was also an increase in embryo mortality at 21°C with >30% mortality of white sturgeon embryos, highlighting a lethal thermal threshold. After hatch, the sublethal consequences continued at 21°C because larval sturgeon exhibited decreased thermal plasticity during CTmax and a dampened transcriptional response during development. Thus, 21°C causes an increase in mortality during embryogenesis with sublethal responses in both whole-animal physiology as well as cellular performance in Nechako white sturgeon embryos and larvae. It is striking that 21°C represents an important thermal threshold in both embryos and larvae across very different sets of traits. This is of concern because the Nechako river has been reaching temperatures in excess of 20°C by the end of June, which persist throughout the summer months (end of September; Environment Canada, 2022).

Our collective evidence suggests temperatures >18°C represent a sublethal limit for multiple phenotypes including transcriptional responses and whole-animal physiology; however, this threshold was not the same for growth. Sturgeon larvae were larger at 21°C than at 18°C, suggesting they may continue to increase their growth rate but at the cost of maintaining their metabolic rate and thermal plasticity. After hatch, larval sturgeon acclimated to 21°C used their yolk-sac faster and were consistently longer than the 14 and 18°C groups, indicating different energy allocation between groups. Maintaining growth despite stressful temperatures is demonstrated across sturgeon species (Earhart *et al*., unpublished data; Hung *et al*., 1993; Allen *et al*., 2006; Kappenman *et al*., 2009; Boucher *et al*., 2014; Poletto *et al*., 2018; Bugg *et al*., 2020; Brandt *et al*., 2022) and may indicate the importance of growth during early-life stages to avoid predation and ultimately to reach maturation size (Bjornson *et al*., 2020). In any case, the sublethal thermal limit for growth seems to be higher than the limits for the other metrics measured in this study; however, this comes at a cost.

Plasticity of the transcriptional response is key in responding to changing environments like warming (Wellband and Heath, 2017; Earhart *et al*., Unpublished data), but acclimation to these temperatures at an early developmental stage decreases this necessary plasticity in white sturgeon. Here, we measured the mRNA levels of 12 different genes, and all of them have an inverted U-shape at control measurements; however, they are not always statistically significant. Conversely, after CTmax, there is little difference in how groups respond, suggesting acclimation did not influence the transcriptional response of these genes after acute thermal stress. However, the patterns seen after acclimation only demonstrate a decrease in cellular performance with increasing temperature because the transcriptional response in 21°C-acclimated fish is diminished. This difference is also evident in the mRNA PCA, where the 14 and 21°C acclimation groups were close together, but the 18°C group is separated. Together, these results support 18°C as a thermal boundary and 21°C as a sublethal temperature for larval white sturgeon mRNA transcriptional activity. This consistent pattern across all genes characterized suggests there may be an active, temperature-sensitive transcriptional control mechanism influencing the abundance of these mRNAs. A control mechanism, such as DNA methylation, which has recently been demonstrated to change in response to thermal acclimation across sturgeon species (Earhart *et al*., unpublished data; Bugg *et al*., 2022; Penny *et al*., 2023). The inverted-U response has also been observed in other species of fish, identified through reduced mRNA transcription in response to increasing temperature, signifying a reduction in cellular performance may be common among fishes undergoing thermal stress (Jeffries *et al*., 2018; Bugg *et al*., 2020, 2022; Mackey *et al*., 2021; Stavrakidis-Zachou *et al*., 2021). The reduction in transcriptional plasticity in response to acclimation to 21°C is likely playing a role in the reduced physiological performance also demonstrated by the larval sturgeon. Not only do we see an inverted U-shape response in the mRNA levels, but this is also detected at the whole-animal level through measurements of whole-embryo metabolism and larval thermal plasticity (ARR).

The white sturgeon embryos increased their metabolic rate between the 14 and 18°C acclimation groups, but did not increase from 18 to 21°C. This suggests the embryos could not further increase their metabolic rate despite acclimation to a warmer temperature, highlighting a sublethal threshold for embryonic metabolism. Because embryos have metabolically intensive developmental demands (Dahlke *et al*., 2020), a suppression or decrease in metabolic rate can aid in identifying these sublethal temperatures because decreases in metabolic rate are usually a survival tactic to endure above-optimum temperatures. Fishes typically suppress their metabolic rate as a mechanism to survive in challenging environments, and this is observed when temperatures begin to reach sublethal and lethal levels (Richards, 2010; McBryan *et al*., 2013; Jeffries *et al*., 2018). The metabolic suppression demonstrated in this study has also been identified in other subarctic Canadian sturgeon populations that were acclimated to 20 and 24°C, suggesting a common metabolic response to these elevated temperatures for northern sturgeons (Bugg *et al*., 2020, 2022).

However, even at their temperature thresholds, the larval sturgeon in this study demonstrated remarkable plasticity across levels of biological organization to thermal acclimation during their most sensitive life stages. Although larvae at 21°C had decreased plasticity compared with fish at 18°C, the acclimation capacity (measured as ARR) at either temperature is much more impressive than what is reported in other fish species (Gunderson and Stillman, 2015; Morley *et al*., 2019). The ARRs reported here, 1.4 (between 14 and 18°C) and 0.9 (between 18 and 21°C), are twice as high compared with other fishes (Morley *et al*., 2019) and thus highlights the outstanding ability of larval white sturgeon to accrue thermal tolerance through acclimation. In fact, across North American sturgeon species, there are reports of impressive increases in thermal tolerance as demonstrated by relative elevation of CTmax after thermal acclimation (Wilkes, 2011; Zhang and Kieffer, 2014; Bard and Kieffer, 2019; Rodgers *et al*., 2019; Bugg *et al*., 2020; Penman *et al*., 2023; Earhart *et al*., Unpublished data). This large acclimatory capacity of sturgeons compared with other fishes may be a result of having large genomes regulating their thermal plasticity, and this would greatly benefit an ancient, long-lived species (Fontana *et al*., 2004; Ellis *et al*., 2014; Bugg *et al*., 2020).

In addition, in this study we observed impressive thermal plasticity in white sturgeon yolk-sac larvae because they demonstrated the highest reported ARR of any sturgeon species or life stage, emphasizing a potential role for development to impact thermal plasticity because ARR likely decreases with age (Wilkes, 2011; Bard and Kieffer, 2019; Bugg *et al*., 2020; Penman *et al*., 2023; Earhart *et al.*, in review). This increased plasticity observed during early life, in which exposure to high temperatures during critical developmental windows can cause long-lasting impacts on phenotype, will ideally make the fish more suited for its future environment (Burggren and Mueller, 2015; Burggren, 2020; Earhart *et al.*, 2022a). This type of plasticity may be especially important for long-lived species with long generational times like white sturgeon because they must rely heavily on plastic physiological and molecular responses to cope with a changing environment rather than adaptation through rapid generational time. There is a limit, however, to this acclimatory capacity and subsequent plasticity. In many northern sturgeon species, decreases in thermal plasticity in these populations are often observed as temperatures reach 20°C (Zhang and Kieffer, 2014; Bugg *et al*., 2020; Penman *et al*., 2023), possibly indicating an innate thermal threshold for beneficial plasticity (Earhart *et al.*, 2022a).

Indeed, acclimation to warm temperatures near an organism’s thermal limits affects traits like thermal performance and plasticity (McBryan *et al*., 2013; Little *et al*., 2020; Earhart *et al.*, 2022a). As expected, acclimation to warmer temperatures did increase thermal tolerance (Morley *et al*., 2019) in the larval sturgeon, but the extent of plasticity induced by warm acclimation was lower as temperature increased. The ARR between 18 and 21°C was 0.5 lower than between 14 and 18°C, demonstrating a temperature threshold for thermal plasticity, although these ARRs are both still higher than what is reported in other species. A decrease in thermal plasticity after acclimation to temperatures past the optimum has been observed across fish species and suggests that there is a hard limit to thermal plasticity through acclimation capacity (Seebacher *et al*., 2015; Rohr *et al*., 2018; Morgan *et al*., 2020; Earhart *et al.*, 2022a). Our findings indicate that in white sturgeon, a species with delayed maturity and long generation times, long-term acclimation to warming river temperatures will not increase plasticity indefinitely to compensate for the rapidly warming environments they inhabit.

The sublethal thresholds demonstrated here for Nechako white sturgeon embryos and larvae highlight the need to reassess the summer temperature monitoring program for the Nechako river. Based on the temperatures investigated in this study, where fish reared at 18°C were less negatively affected than those at 21°C, it raises the question whether 20°C is an appropriate threshold for white sturgeon. Additional studies are needed to assess the long-term impacts of warming temperature during development because even 18°C could have negative physiological impacts later in life. Furthermore, the current 20°C threshold has been breached by 2°C in recent years, such that the Nechako river will reach 22°C during the middle of the summer. To accommodate for when temperatures surpass regulatory thresholds and to create a more inclusive, holistic monitoring program for the Nechako that will protect both salmonids and sturgeons, we should consider lowering the temperature threshold <20°C to 18°C.

Lowering the limit to 18°C would help with embryo survival and also help the larval sturgeon develop their phenotypic plasticity, which is of crucial importance. Importantly, this lowered threshold in the Nechako to help young-of-the-year sturgeon and spawning salmon is crucial because both species have reduced physiological performance at temperatures >18°C (Macdonald *et al*., 2012; Levy and Nicklin, 2018). Finally, temperature monitoring should start in the beginning of June rather than July because the sturgeon are most vulnerable during this time, and climate change is contributing to shifts in seasonality while also continuing to rapidly increase the rate and intensity of temperature change. As a critically endangered species with complete and continuing recruitment failure, ensuring the Nechako white sturgeon have suitable temperatures during the summer reproductive and developmental months may help encourage larval survival and reduce one of the many stressors they must overcome to successfully spawn, survive and ultimately recruit future generations.

## Funding

This research was supported by an NSERC Discovery grant (RGPIN-2017-04613) and a Canada Research chair (CRC-2021-00040) to P.M.S., an NSERC Collaborative Research and Development grant (CRDPJ 523640–18) to C.J.B., an NSERC Discovery grant to D.W.B. (RGPIN-2017-06895), and an NASPS research travel grant to M.L.E.

## Data availability

Data for this study can be found on dryad data depository:

Dryad, Dataset, https://doi.org/10.5061/dryad.zw3r228cr

## Author contributions

M.L.E., R.J.P. and C.J.B. conceived and designed the experiments. M.L.E., P.R.M., T.S.B., R.J.P. and N.S. executed experiments and completed fish care. M.L.E. analysed all data. P.M.S. and D.W.B. assisted in data interpretation. M.L.E. drafted the manuscript, and all authors read and edited.

## Supplementary Material

Web_Material_coad032
