# Supplementary material for: Identification of upper thermal thresholds during development in the endangered Nechako white sturgeon with management implications for a regulated river
Source: Conserv Physiol. 2023 May 23;11(1):coad032. doi: 10.1093/conphys/coad032 (PMC10205467; doi:10.1093/conphys/coad032)
Supplement: Web_Material_coad032 [file web_material_coad032.zip › supplemental_materials_2-4_embryo.pdf]

Supplementary materials

Identification of upper thermal thresholds during development in the endangered Nechako white sturgeon with management implications for a regulated river

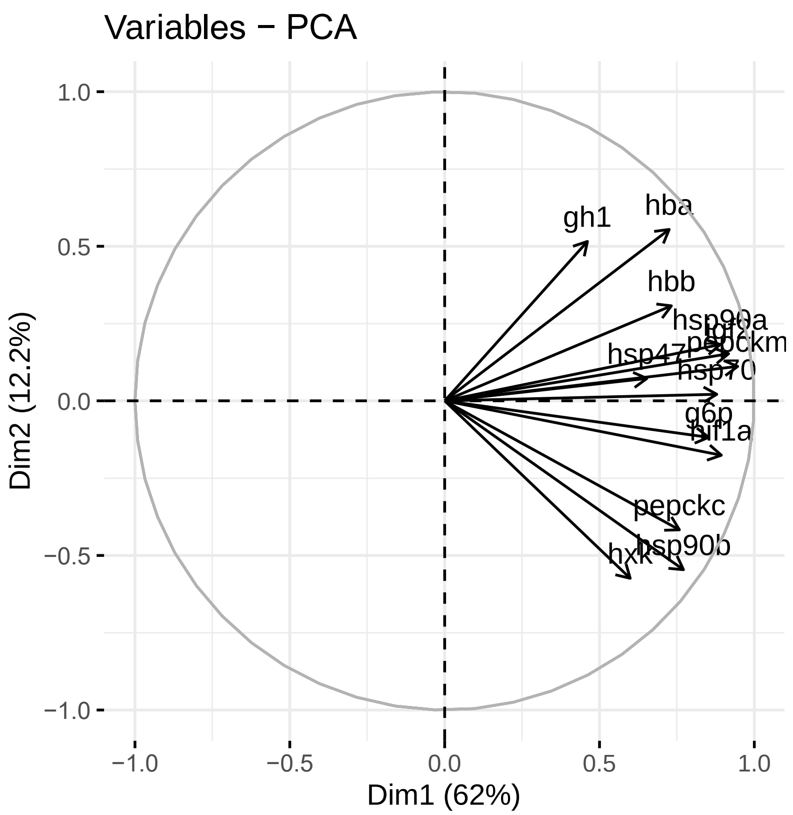

Control		
	Dim.1	Dim.2
hsp70	9.588991	0.02929975
hsp90a	9.823972	2.09974794
hsp90b	7.38315	18.7529545
hsp47	5.298096	0.33311529
hbb	6.654357	5.94058397
igf2	10.43764	1.46965643
g6p	9.035244	0.87916891
pepckc	7.134015	10.9576338
pepckm	11.112754	0.7810766
hxk	4.457673	20.7309845
hba	6.524955	19.3478132
gh1	2.636555	16.7346943
hif1a	9.912598	1.94327088

Supplemental 2. Vector plot and dimension loadings for control PCA in white sturgeon yolk sac larvae.

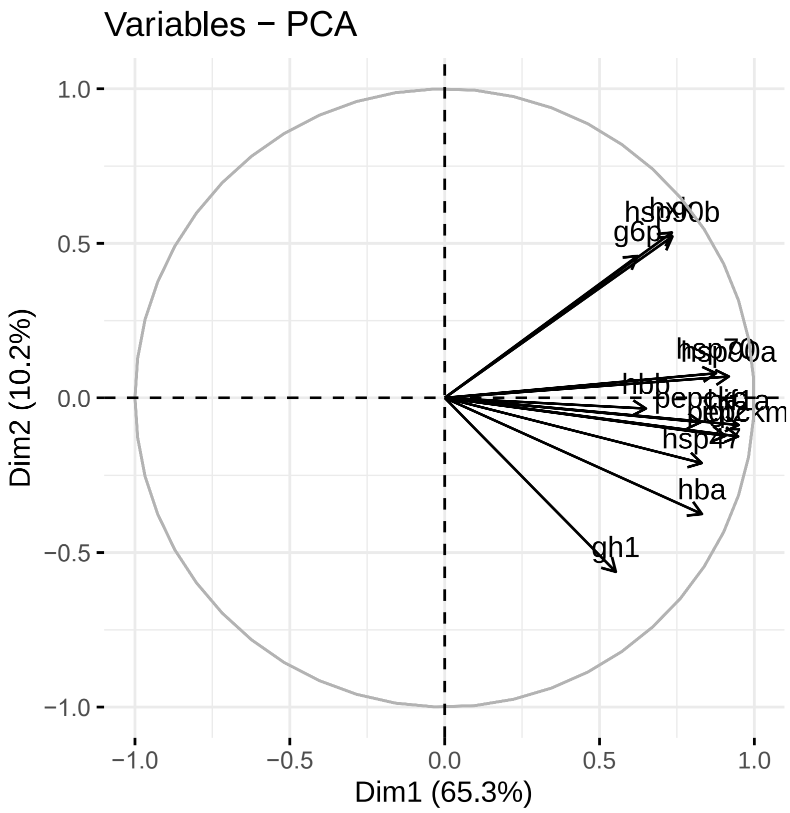

CTMax		
	Dim.1	Dim.2
hsp70	9.037641	0.48205551
hsp90a	9.919252	0.36575406
hsp90b	6.374188	20.5132651
hsp47	8.121927	3.35031841
hbb	4.978315	0.08752868
igf2	9.681218	1.12985768
g6p	4.578818	15.8986585
pepckc	8.056899	0.46256266
pepckm	10.577474	1.17026433
hxk	6.337081	21.5590084
hba	8.124274	10.6260314
gh1	3.594691	23.7761244
hif1a	10.618221	0.57857085

Supplemental 2.Vector plot and dimension loadings for CTmax PCA in white sturgeon yolk sac larvae.

Supplementary materials

Identification of upper thermal thresholds during development in the endangered Nechako white sturgeon with management implications for a regulated river

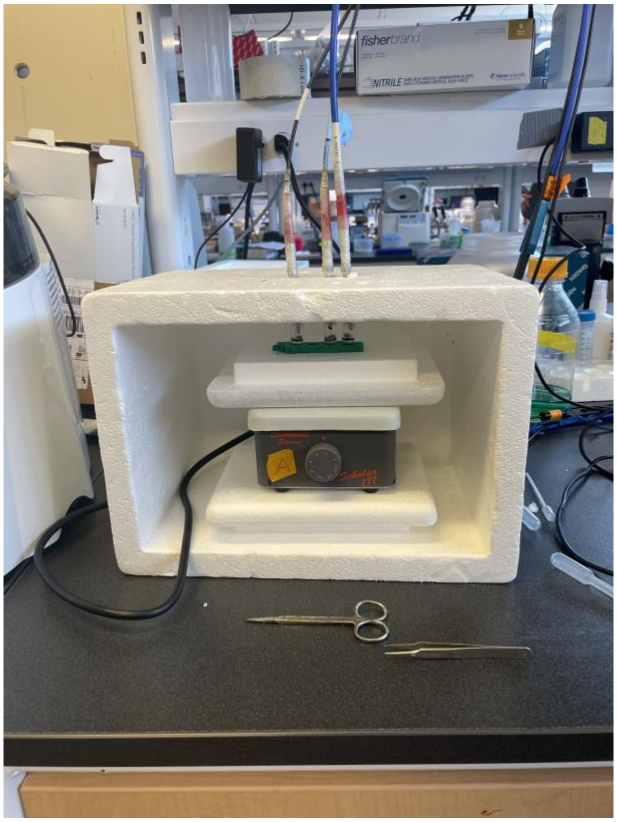

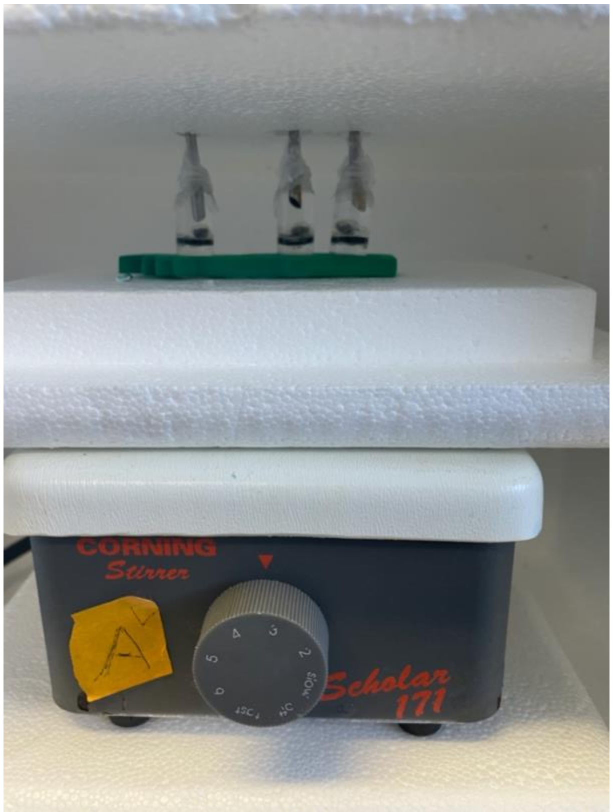

Supplemental 3. MO2 chambers for measuring embryo metabolic rate. Picture on the left shoes entire design, picture on the right shows a single embryo in each MO2 chamber.

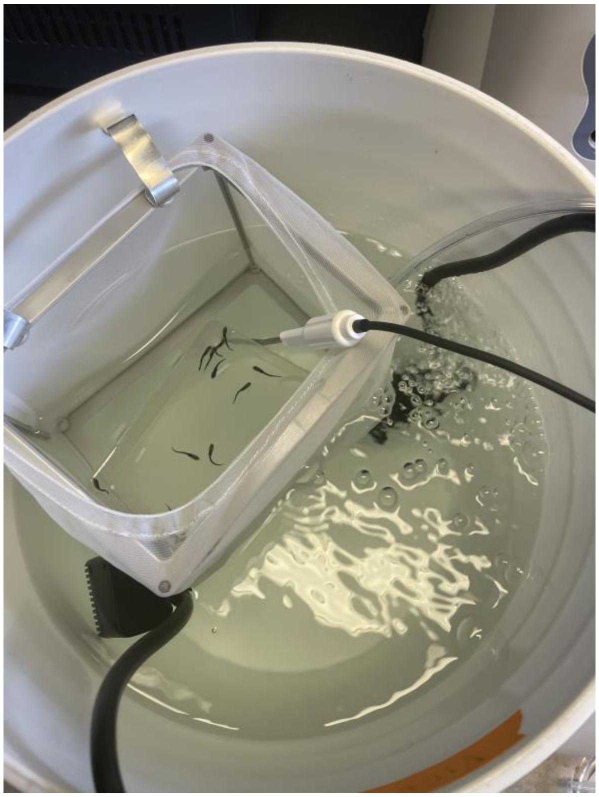

Supplemental 3. CTMax set up for measuring thermal tolerance in white sturgeon yolk sac larvae. Sturgeon are held in a breeder net, inside a bucket filled with oxygenated, dechlorinated water. There are multiple pumps circulating water, heater sticks, an air stone, and thermometers.

Supplementary materials

Identification of upper thermal thresholds during development in the endangered Nechako white sturgeon with management implications for a regulated river

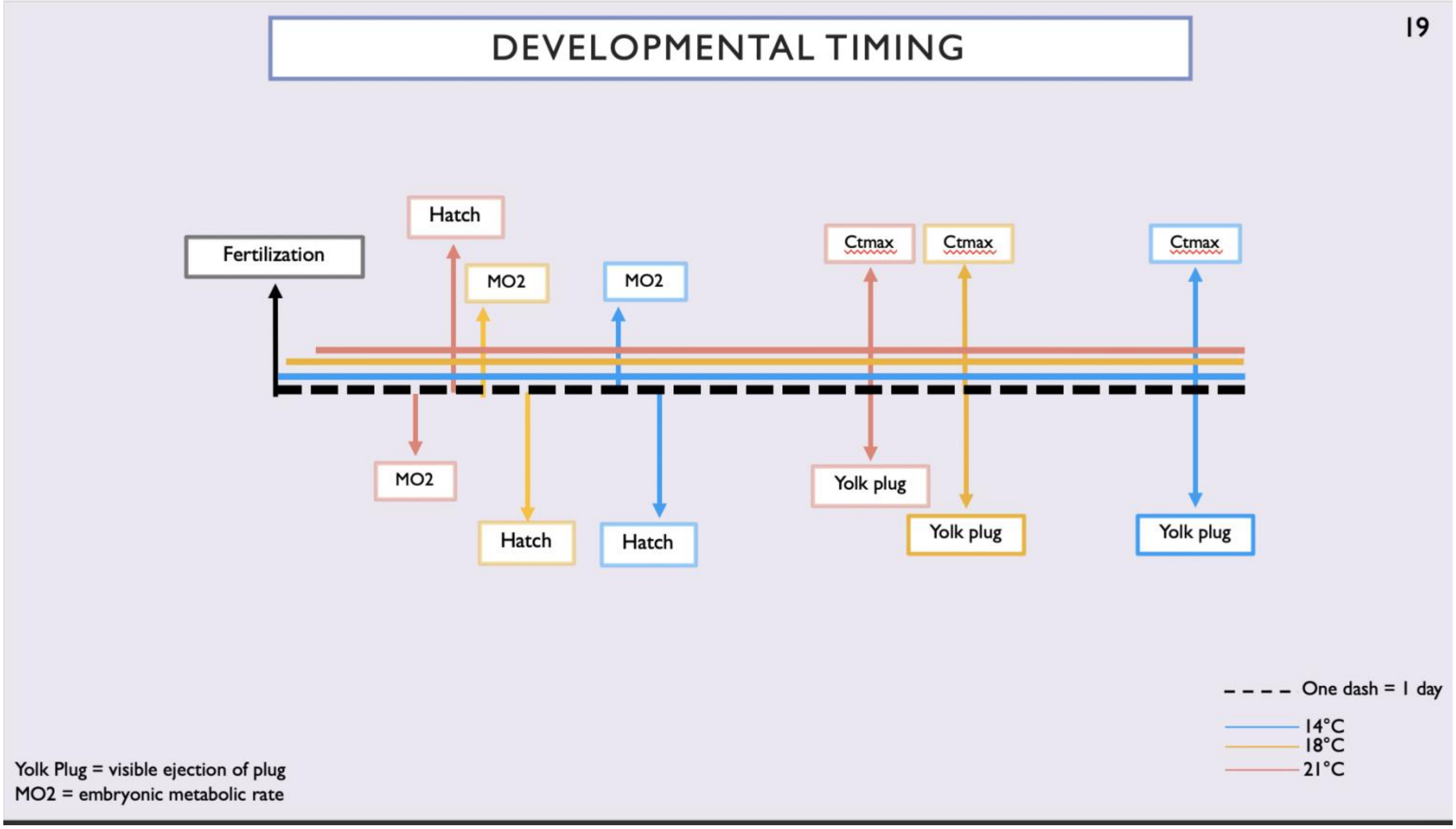

Supplemental 4. Developmental timing over days for each incubation temperature in white sturgeon embryos and yolk sac larvae. One dash = one day.
